# SERVICE LEARNING TO INCREASE EXERCISE IN OLDER ADULTS: TESTING A GROWTH MINDSET INTERVENTION

**DOI:** 10.1093/geroni/igz038.1976

**Published:** 2019-11-08

**Authors:** Abby L Heckman Coats, Zhaoming Wang, Therese Miller

**Affiliations:** Westminster College, Fulton, Missouri, United States

## Abstract

Exercise is one of the best ways to maintain psychological and physical health in late life (Kohl et al., 2012). However, many older adults experience barriers to developing an exercise routine, such as fear/anxiety, limited perceived control, and lack of self-efficacy (Bock et al., 2014). Recently, successful psychological interventions to encourage exercise have been demonstrated (Brothers & Diehl, 2017; Lachman et al., 2018). Inspired by this success, we developed a service learning activity to address this community-based problem. Undergraduate psychology students visited local senior centers. During the visits, the students implemented an intervention to increase physical activity in older adults. Traditionally-aged undergraduate students ate lunch with the older adults and engaged in informal discussions. Then, after establishing rapport, they implemented an exercise-boosting intervention to the experimental group. (A control group did not receive the intervention.) The older adults in the experimental group heard quotes from peers explaining how they had overcome barriers to exercise. The peers’ stories included growth-mindset language (Yeager, Paunesku, Walton, & Dweck, 2013), such as an initial reluctance to exercise, followed by hard work and finally implementing a successful exercise regimen. Then the older adults wrote their own letters to a peer, explaining how they have overcome barriers in the past and how they could continue to do in the domain of exercise. This activity was helpful for both the older adults and the undergraduate students, as documented by qualitative interviews and questionnaire responses. The students reported more positive attitudes about aging after the activity.

